# Temporal hemodynamic classification of two hands tapping using functional near—infrared spectroscopy

**DOI:** 10.3389/fnhum.2013.00516

**Published:** 2013-09-02

**Authors:** Nguyen Thanh Hai, Ngo Q. Cuong, Truong Q. Dang Khoa, Vo Van Toi

**Affiliations:** ^1^Biomedical Engineering Department, International University of Vietnam National Universities in Ho Chi Minh CityHo Chi Minh City, Vietnam; ^2^Department of Electronics and Telecommunications, Faculty of Electrical and Electronics Engineering, University of Technical Education HCMCHo Chi Minh City, Vietnam

**Keywords:** polynomial regression algorithm, support vector machines, artificial neural networks, hand tapping recognition, functional Near-Infrared Spectroscopy

## Abstract

In recent decades, a lot of achievements have been obtained in imaging and cognitive neuroscience of human brain. Brain's activities can be shown by a number of different kinds of non-invasive technologies, such as: Near-Infrared Spectroscopy (NIRS), Magnetic Resonance Imaging (MRI), and ElectroEncephaloGraphy (EEG; Wolpaw et al., 2002; Weiskopf et al., 2004; Blankertz et al., 2006). NIRS has become the convenient technology for experimental brain purposes. The change of oxygenation changes (oxy-Hb) along task period depending on location of channel on the cortex has been studied: sustained activation in the motor cortex, transient activation during the initial segments in the somatosensory cortex, and accumulating activation in the frontal lobe (Gentili et al., 2010). Oxy-Hb concentration at the aforementioned sites in the brain can also be used as a predictive factor allows prediction of subject's investigation behavior with a considerable degree of precision (Shimokawa et al., 2009). In this paper, a study of recognition algorithm will be described for recognition whether one taps the left hand (LH) or the right hand (RH). Data with noises and artifacts collected from a multi-channel system will be pre-processed using a Savitzky–Golay filter for getting more smoothly data. Characteristics of the filtered signals during LH and RH tapping process will be extracted using a polynomial regression (PR) algorithm. Coefficients of the polynomial, which correspond to Oxygen-Hemoglobin (Oxy-Hb) concentration, will be applied for the recognition models of hand tapping. Support Vector Machines (SVM) will be applied to validate the obtained coefficient data for hand tapping recognition. In addition, for the objective of comparison, Artificial Neural Networks (ANNs) was also applied to recognize hand tapping side with the same principle. Experimental results have been done many trials on three subjects to illustrate the effectiveness of the proposed method.

## Introduction

Human brain has a complex structure with around 100 billion neurons, so it is a big challenge for all scientists in biological computing (Wolpaw et al., 2002). These neurons can communicate from one to another with or without external excitations to make typical decisions (pattern recognition, cognition, motion, and others; Critchley, 2009). Moreover, in prefrontal cortex of human brain plays an important role in social activity for both adults and children. Tobias Grossmann represented a review related to the role of prefrontal cortex of human brain, in which specific areas in the adult human brain as social brain could process the social world (Aydore et al., 2010; Grossmann, 2013) and also Tila Tabea Brink et al. investigated about orbitofrontal cortex in children with 4− to 8-year-old through processing empathy stories (Brink et al., 2011). The result is that children could passively follow these stories presenting social situations. Regarding prefrontal cortex, EEG electrodes were mounted on frontal positions of human brain for wheelchair control (Ahmed, 2011). In particular, user could move eyes to drive the electrical wheelchair to reach the desired target.

In recent decades, a lot of achievements have been obtained in imaging and cognitive neuroscience of human brain. Brain's activities can be explored using different kinds of non-invasive technologies, such as: Magnetic Resonance Imaging (MRI), Near-Infrared Spectroscopy (NIRS), and ElectroEncephaloGraphy (EEG; Wolpaw et al., 2002; Weiskopf et al., 2004; Blankertz et al., 2006; Ince et al., 2009). Many researchers have been attracted by these technologies with many approaches to find out problems related to human brain for rehabilitation and treatment. For the rehabilitation problem, information obtained from human brain using EEG technique could be employed to perform shared control of motion wheelchairs (Tanaka et al., 2005). A brain simulator can lead to improve or to recover the cognitive/motor functions of tetraplegic patients with degenerative nerve diseases spinal cord injuries (Kauhanen et al., 2006). In these non-invasive technologies, the NIRS technology is often applied to measure Oxygen Hemoglobin (Oxy-Hb), deOxy-Hb, and Total-Hb concentration changes. These changes allow us predict brain activations related to body behaviors.

fNIRS has become the convenient technology for experimental brain purposes. This non-invasive technique emits near infrared light into the brain to measure cerebral hemodynamics as well as to detect localized blood volume and oxygenation changes (Tsunashima and Yanagisawa, 2009). The change of oxy-Hb along task period depending on the location of channels the cortex has been studied: sustained activation in the motor cortex, transient activation during the initial segments in the somatosensory cortex, and accumulating activation in the frontal lobe (Gentili et al., 2010). Oxy-Hb concentration at the aforementioned sites in the brain can also be used as a predictive factor allows prediction of subjects' investigation behavior with a considerable degree of precision (Shimokawa et al., 2009).

fNIRS technique is a non-invasive technique which is applied to monitor human body for diagnosis and treatment (Bozkurt et al., 2005; Macnab et al., 2011; Reher et al., 2011). Hiroshi Taniguchi et al. investigated six subjects with unilateral spatial neglect (USN)-positive (+) and 6 others with USN-negative (Taniguchi et al., 2012). In this research, brain activity was simulated by prism adaptation tasks using fNIRS. The result showed that there was a typically great reduction in Oxy-Hb of the USN (+). For monitoring carotid endarterectomy, one was applied the NIRS technique to evaluate its reliability in the detection of clamping ischemia (Pedrini et al., 2012). The result found that there were three patients who represented transient ischemic deficits at awakening and no case of perioperative stroke or death.

In addition, fNIRS technique has been appeared as an alternative brain-based experimental technique (Lloyd-Fox et al., 2010) to measure human thoughts and activities for rehabilitation. For evaluating behaviors related to human brain during experiments, subjects feel free for performing his or her brain activities. In particular, this technique has been successfully used to study brain functions such as assessment of motor task from everyday living, athletic performance, recovery from neurological illness (Hu et al., 2010), assessment of verbal fluency (Schecklmann et al., 2010), and quantification of brain function during finger tapping (Sato et al., 2007). However, to the best of our knowledge, there have been a few applications of the fNIRS technique to quantify the motor control signals leading to brain simulator for rehabilitation (Chunguang et al., 2010; Gentili et al., 2010).

Neural networks can be used for cognition brain tasks as a classification module, in which wavelet decomposition can be used as feature extractions (Khoa and Nakagawa, 2008); wavelet can be used to remove artifacts (Molavi and Dumont, 2010). Base on the slope of straight line, hand side tapping can be distinguished (Ngo et al., 2012). Oxy-Hb and Deoxy-Hb can also be used directly with SVM algorithm for the recognition of hand tapping (Sitaram et al., 2007).

Savitzky–Golay (SG) filters have been used to smooth signals and images with noises as well as artifacts in recent years. In the SG filters, the coefficients of the local least-square polynomial fit are pre-computed to preserve higher movements and then the output of the filter is taken at the center of the window (Savitzky, 1964; Steinier et al., 1972; Gorry, 1990). In this paper, the SG filter was applied to reduce spike noises of Oxy-Hb signals. The Oxy-Hb signals after filtering allow us be easier in recognizing left (LH) or right hand (RH) tapping status. Moreover, a Polynomial Regression (PR) approach has been applied for estimation of signals and images with noise (Cui and Alwan, 2005; Cai et al., 2007; Zhang et al., 2009; Khan et al., 2011). In our research, in order to estimate Oxy-Hb signals, the PR algorithm was used to produce polynomial curves with their features. Based on these features, one can classify tapping hand tasks.

Support Vector Machine (SVM) algorithms have been applied for classification problems in the machine learning community in recent years. In this case, the SVM was employed to classify hypothyroid disease based on UCI machine learning dataset (Chamasemani and Singh, 2011). Another application related to medical images is that the SVM was utilized to recognize the leaf spectral reflectance with different damaged degrees in the image processing and spectral analysis technology (Dake and Chengwei, 2006). In this project, the SVM algorithm (Sitaram et al., 2007) was applied to recognize hand tapping tasks using fNIRS technology. Oxy-Hb signals after reducing noise will be extracted features using a PR algorithm. Based on coefficients obtained from the PR, the SVM algorithm will be applied for the recognition of the LH and RH tapping tasks.

Another algorithm for classification is that a recursive training algorithm for EEG signals using Artificial Neural Networks (ANNs) to generate recognition patterns from EEG signals was proposed to control electric wheelchair (Tanaka et al., 2005; Singla et al., 2011). Mental tasks were classified for wheelchair control using prefrontal EEG (Rifai Chai, 2012). The relevant mental tasks used in this paper are mental arithmetic, ringtone imagery, finger tapping, and words composition with additional tasks which are baseline and eyes closed. The feature extraction is based on the Hilbert Huang Transform (HHT) energy method and then the ANNs with the Genetic Algorithm (GA) optimization (Subasi et al., 2005) was applied for classification. The result is that the accuracy of the proposed classification algorithm with the five subjects participated was between about 76 and 85%.

**Figure 1:**
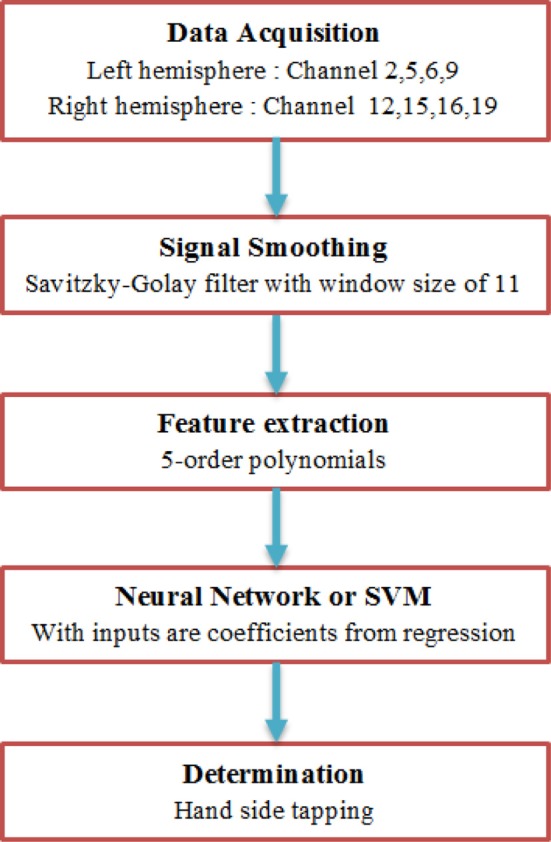
**Recognition algorithm block diagram**. First of all, Savitzky–Golay filter is used to reduce noises as well as artifacts. After that, feature of Oxy-Hb is found by a polynomial regression based on its coefficients. Finally, Artificial Neural Network or Support Vector Machines is used to determine whether left hand or right hand is tapped.

In this paper, we proposed the recognition algorithm for developing a brain computer interface using fNIRS. First of all, Savitzky–Golay filter is used to reduce noises as well as artifacts. Coefficients, which are features of Oxy-Hb signals, are found by using a PR algorithm. For the recognition of tapping hands related to the left and right brain activation, ANN and SVM algorithms were used. These two methods will be compared to find out the best one. The results and discussion about tapping hand activity will be shown to illustrate the effectiveness of the proposed approaches. This process is shown in Figure 1.

## Materials and methods

### Subjects and the experimental setup

**Figure 2:**
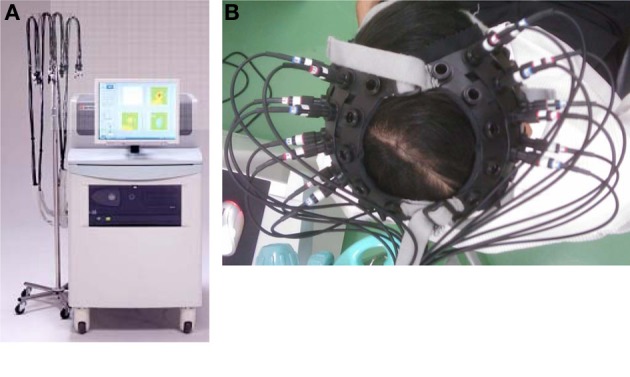
**(A)** fNIRS FOIRE-3000 system. This system operates at three different wavelengths of 780, 805, and 830 nm. **(B)** Subject' head with installed probes. The distance between pairs of emitter and detector probes was set at 3 cm and all probes were attached with holders.

A multichannel fNIRS instrument, FOIRE-3000 (SHIMAZU Co. LTD, Japan), is used to acquire brain Oxy-Hb. This machine was located at Lab-104 of Biomedical Engineering Department, International University, VNU, Vietnam. The FOIRE 3000 system with the eight pairs of the probes, consisting of the illuminator and detector optodes, produces 24 channels as shown in Figure 2A. These probes were placed on the scalp to collect fNIRS data, in which the detectors were installed at a 3 cm distance from the illuminators. The optodes were arranged to install at the left hemisphere on the head of the subject.

Oxy-Hb concentration changes in motor control area of human brain was captured from a set of the holder with 24 channels for both hemispheres using the fNIRS technique as shown in Figure 2B. In particular, when the subject performs one typical activity, brain signals will be obtained from the fNIRS system and then calculated to produce three types of signals [Oxy-Hb (red), Total-Hb (green) and Deoxy-Hb (blue)] corresponding to three wavelengths (780, 805, and 830 nm), in which [Total-Hb] = [Oxy-Hb] + [Deoxy-Hb]. Moreover, the distance between pairs of emitter and detector probes was set at 3 cm and all probes were attached with holders arranged on different sides of human brain hemispheres depending on users. Concentration changes of three signals produce time points in an output. In this research, Oxy-Hb changes are calculated in the following formula (Shimadzu Corporation, 2010):

(1) Oxy=−3.6132∗Abs[780nm]+1.1397∗Abs[805nm]+ 3.0153∗Abs[830nm]

in which Abs: Absorbance.

Three subjects (male, average: 25 years old, 60 kg weights, right-handed) were participated into this study. All participants were healthy and showed no musculoskeletal or neurological restrictions or diseases. Before participating into the experiments, each subject was asked to fill out a questionnaire consisting of patient's identification, age and gender, which was kept confidential. The tenets of the Declaration of Helsinki were followed; the local Institutional Review Board approved the study. These subjects informed consent agreement after reading and understanding of the experiment protocol and the fNIRS technique.

**Figure 3:**
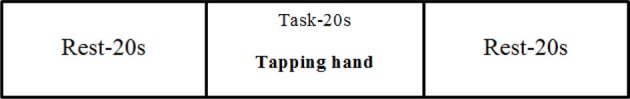
**Setting of experiment protocol**. The subject relaxed in 20 s, tapped his hand up/down about 10 times in 20 s, and then rested 20 s.

After reading and understanding the experiment protocol and the fNIRS technique, he will start doing hand tapping. The subject was required to perform hand tapping motions, both left and right sides as motor activities. In these hand tapping motions, a protocol includes 20 s (Rest)—20 s (Task)—20 s (Rest), it means that the subject relaxed in 20 s, tapped his hand up/down about 10 times in 20 s, and then rested 20 s, as shown in Figure 3.

**Figure 4:**
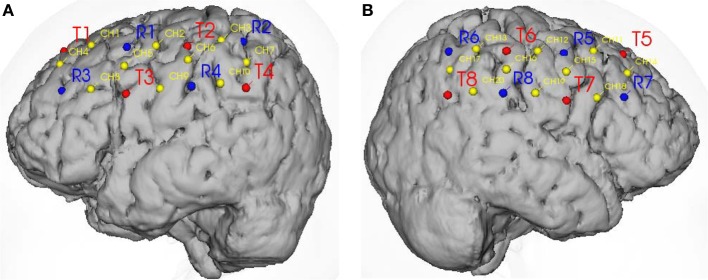
**Probes location and channels on two hemispheres. (A)** Probes location (red—emitter, blue—detector) and channels on the motor control area of the left hemisphere. **(B)** Probes location and channels (yellow) on the motor control area of the right hemisphere.

Oxy-Hb data were collected on 20 channels, in which 10 channels are of the left brain side and that of the opposite side will be obtained for hand tapping recognition. However, we just chose 4 channels of each side which focus on hand and leg motion area to analyze and to estimate features. In particular, the left brain channels are 2, 5, 6, 9, and the 12, 15, 16, 19 channels are of the right brain side as in Figures 4A,B. In this research, Oxy-Hb data obtained from these channels will be processed to recognize hand tapping tasks. Without loss of generality, the natural architecture is different from person to person. The probes are allocated on the holder, in which the transmitter probes and receiver probes are predicted to cover as much as area of brain based on the physical structure of each subject. The authors (Aihara et al., 2012) combined EEG and NIRS for estimation of cortical current source. The probes position using stylus marker to allow co-registration of EEG and NIRS results. In this paper, we also used marker to find out the average positions of motor area of human brain cortex. To achieve more accuracy, the NIRS activity was mapped onto cerebral cortex using fusion software (Shimadzu Corporation, 2010). From this evidence, we proposed the selection of channels 2, 5, 6, 9 and 12, 15, 16, 19 for hand tapping recognition with the 20-channel NIRS system configured above.

### Data pre-processing

Brain data of a subject acquired from the channels have noise and artifacts. In order to obtain more smoothly brain data, a filter as the Savitzky–Golay filter was applied in this paper. The Savitzky–Golay filters (Orfanidis, 2010) are also known as polynomial smoothing. It means that the idea of the polynomial smoothing is replacing samples of signal by the values that lie on the smoothing curve. In moving an average FIR filter, the output is a simply average version of its inputs, in which this filter has the response of the low-pass filter. In practice, NIRS signals fluctuate along time corresponding to excitations and have the unknown specific frequency. Therefore, it could not be the average of inputs with the arbitrary FIR filter length. In this research, to track the acquired signal, the Savitzky–Golay filter as the FIR filter can be used.

In general, we can evaluate a polynomial with the order of *d* to smooth the length-*N* data *x* with the condition *N* ≥ *d* + 1. Assume that, the data *x* is the type of a vector

(2) $$\text{x}={\left[x_{-M},\ldots ,x_{-1},x_{0},x_{1},\ldots ,x_{M}\right]}^{T}$$

in which *N* samples of *x* are replaced by the polynomial with the order of *d* as follow:

(3) $${\hat{x}}_{m}=c_{0}+c_{1}m+\cdots +c_{d}m^{d},\;-M\leq m\leq M$$

where *c*_0_, *c*_1_, …, *c*_*d*_ denote polynomial coefficients. *M* is the number of points on either side of *x*_0_

In this case, there are *d* + 1 based on the vector *s*_*i*_, *i* = 0, 1, …, *d* as follows:

(4) $$s_{i}(m)=m^{i},\;-M\leq m\leq M$$

Thus, we can write the vector *S* as follows:

(5) $$S=\left[s_{0},s_{1},\ldots ,s_{d}\right]$$

in which s_0_, s_1_, …, s_*d*_ are the polynomial basic vectors.

The smooth values in (3) can be re-written in the following equation:

(6) $$\hat{x}=\sum_{i=0}^{d}c_{i}s_{i}=[s_{0},s_{1},\ldots ,s_{d}]\;[\begin{array}{c} c_{0}\\ c_{1}\\ \vdots \\ c_{d} \end{array}]=Sc$$

Coefficients of the desired filters are obtained as follows:

(7) $$B=SG^{T}=GS^{T}=SF^{-1}S^{T}=\left[b_{-M},\ldots ,b_{0},\ldots b_{M}\right]$$

in which, *b*_−*M*_, …, *b*_0_, …, *b*_*M*_ are the column filters of the Savitzky–Golay filter set.

(8) $$\{\begin{array}{l} F=S^{T}S\\ G=SF^{-1} \end{array}$$

Finally, the values to create more smoothly signals are estimated in the following equation:

(9) $${\hat{x}}_{m}=b_{m}^{T}x,m=-M,\ldots ,0\ldots ,M$$

in which, *b*^*T*^_*m*_ are the transpose version of *b*_*m*_.

In this paper, the Savitzky–Golay filter will be utilized to smooth spikes of brain Oxy-Hb signals for identifying hand tapping tasks. The filtered Oxy-Hb signals allow us extract features with reliable information.

### Feature extraction

In general, the first step in classification work is to find the features of data samples. For this purpose, there are many methods such as Principle Component Analysis (PCA), Independent Component Analysis (ICA) and etc. However, hemodynamic response of human brain changes in time domain. Moreover, we want to evaluate the Oxy-Hb concentration corresponding to hand tapping tasks based on analyzing numeric as well as having a look in graphical figures.

PR algorithm (Montgomery and Runger, 2003) presents the relationship between amplitude and time of a signal. In this paper, the PR algorithm was applied to analyze brain Oxy-Hb data in blood flow corresponding to hand tapping tasks. From the processed data, one can distinguish the difference between the LH and RH tapping times corresponding to the difference of the Oxy-Hb concentration changes.

Assumed that we have the set of two-dimensional data, (*x*_1_, *y*_1_), …, (*x*_*n*_, *y*_*n*_), where each of *x* and *y* has no information about the other. Our problem is fitting a polynomial curve generated by a typical data. Thus, the relationship between *x* and *y* can be found out. Based on the coefficients of the regression curve with the order of 5, one can estimate the hand tapping. In particular, the PR equation between independent variable *x* and *y* fitted can be expressed as:

(10) $$\hat{y}={\hat{h}}_{0}+{\hat{h}}_{1}x+{\hat{h}}_{2}x^{2}+\cdots +{\hat{h}}_{m}x^{m}$$

in which, ${\hat{h}}_{0},\;{\hat{h}}_{1},\;{\hat{h}}_{2},\;\cdots ,\;{\hat{h}}_{m}$ are estimated values of *h*_0_, *h*_1_, *h*_2_, …, *h*_*m*_. There are *m* regressors and *n* observations, (*x*_*i*1_, *x*_*i*2_, …, *x*_*im*_, *y*_*i*_), *i* = 1, 2, …, *n* corresponding to (*x*_*i*_, *x*^2^_*i*_, …, *x*^*m*^_*i*_, *y*_*i*_). In this equation, the powers of *x* play the role of different independent variables.

The PR model can be re-written as a system of linear equations

(11) y=Xh+ε

where: ε = [ε_1_, ε_2_, …, ε_*n*_]^*T*^ is a vector of error.

The ordinary least square $\hat{h}$ of *h* given by the arguments that minimize the residual sum of squares and the distributive law is employed. One obtains the equation,

(12) $$\text{RSS}(h)=y^{\prime }y+h^{\prime }(X^{\prime }X)h-2y^{\prime }Xh$$

Equation 12 is minimized by taking $\frac{\partial \text{RSS}}{\partial h}$ and set the result to zero. This leads to

(13) $$X^{\prime }Xh=X^{\prime }y$$

The ordinary least square in the case of the inverse of *X*′*X* exists is given by

(14) $$\hat{h}={\left(X^{\prime }X\right)}^{-1}X^{\prime }y$$

**Figure 5:**
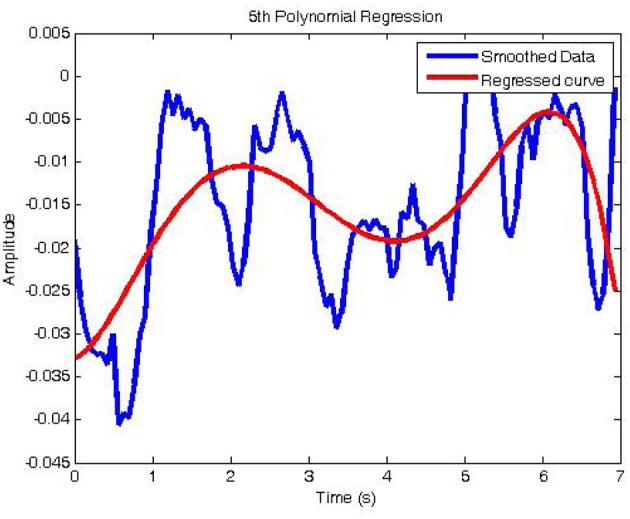
**The regression signal of filtered channel 2**. Sudden changes had been removed with the window size of 11.

From these coefficients, one can determine problems of the LH tapping or RH tapping tasks with the measured brain data using the fNIRS technology. Figure 5 represents the regressed signal of the channel-2 corresponding to Equation 15. Similarly, the regression signals of channels 5, 6, 9, 12, 15, 16, and 19 can be shown.

(15) $$y_{C2}=-0.0001x^{5}+0.0023x^{4}-0.0114x^{3}+0.0182x^{2}+0.0043x-0.0329$$

In this figure, the blue Oxy-Hb signal after the Savitzky–Golay filter was calculated to produce the red regressive curve. Each hand tapping creates the regressive Oxy-Hb curves which contain information or its feature coefficients. For recognition of hand tapping types, these coefficients will be given the input of the identification system or called the identification algorithms.

The regressed polynomial must represent the original signal with the best fit. The smaller error between the origin (here is the filtered NIRS signal) and the regressed signal is higher than the order of the polynomial is. It means that one should choose the order not only to fit the origin but also to show the general trend and the characteristic of NIRS signal. In practice, the NIRS signal can not change immediately at the moment of tapping hand. For example, one hand moving up or down will make an excitation to both hemispheres. Therefore, in 20 s of tapping hand, one person could take 10 times of moving hand up and down. In this case, Oxy-Hb level, which will flow from the lowest to highest level in short time, is not the “trend” of overall signal. This is the reason for choosing the polynomial with the order of 5.

### Artificial neural network

**Figure 6:**
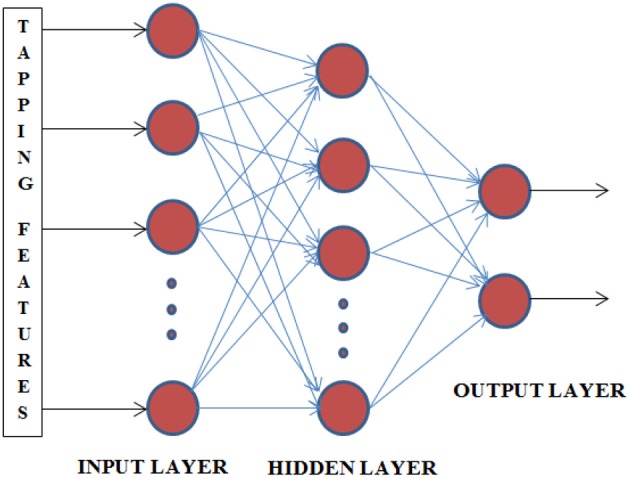
**Architecture of classification network**. This net has 48 nodes input, 100 nodes at hidden layer and 2 nodes at output.

ANNs are the very powerful tools for the problems of classification and pattern recognition. We can use the estimated coefficients as the features from the PR algorithm by connecting with a multilayer feed forward network for recognition. The architecture of this network used here consists of an input layer, one hidden layer, and the output layer as shown in Figure 6. In particular, input samples are the features from channel coefficients corresponding to Oxy-Hb concentration changes. The number of hidden nodes is carefully chosen for this case to obtain higher performance. Therefore, it can be chosen as an average of number of the input nodes and the output nodes. With the hidden layer, we used the double sigmoid function and this sigmoid function was also used for the output layer.

In general, standard back propagation is used for training the network with three layers. It is a gradient descent algorithm, in which the network weights are moved along the negative of the gradient of the performance function. With this argument, the training is based on the minimization of the following error function:

(16) $$E=\sum_{n=1}^{N}{\left(o_{n}-d_{n}\right)}^{2}\text{​},$$

where *N* is number of samples, *o* is network output and *d* is desired output.

Suppose that the network has *I* nodes of the input layer, *J* nodes of the hidden layer and the output layer is *K* nodes. Call *w*^(1,0)^_*j, i*_ is weight from the *i*th node of the input layer to the *j*th node of the hidden layer and *w*^(2,1)^_*k, j*_ is weight from the *j*th node of the hidden layer to the *k*th node of the output layer. The backpropagation learning of the 3-layers network is shown in Table 1. The application is that with the LH tapping, the output is desired to get the value of [1; 0] and [0; 1] is the desirable value of the right tapping. The ANN is one of the approaches which is often used for recognition. In this research, the SVM is also applied to identify hand tapping tasks through Oxy-Hb flowing in brain blood.

**Table 1:** **The three-layers network with backpropagation learning**.

Random initial weights
While the Mean Square Error (MSE) is unsatisfied or the number of epochs is not exceed,
For each input *x*_*p*_, 1 ≤ *p* ≤ *P*, (^*^)
Compute the inputs of hidden layer net^(1)^_*p, j*_;
Compute the outputs of hidden layer *x*^(1)^_*p, j*_;
Compute the inputs of ouput layer net^(2)^_*p, k*_;
Compute the outputs of network *o*_*p, k*_;
Modify outer weights
Δ*w*^(2, 1)^_*k, j*_ = η (*d*_*p, k*_ − *o*_*p, k*_)*S*′(net^(2)^_*p, k*_)*x*^(1)^_*p, j*_
Modify weights between input layer and hidden layer
$\Delta w_{j,\;i}^{\left(1,\;0\right)}=\eta \sum_{k=1}^{k}\left(\left(d_{p,\;k}-o_{p,\;k}\right)S^{\prime }\text{​}\left({\text{net}}_{p,\;k}^{\left(2\right)}\right)w_{k,\;j}^{\left(2,\;1\right)}\right)S^{\prime }\text{​}\left({\text{net}}_{p,\;j}^{\left(1\right)}\right)x_{p,\;i}$
End (^*^)
End While
Where: *S*() is the active function, η is the learning rate.

### Support vector machines

**Figure 7:**
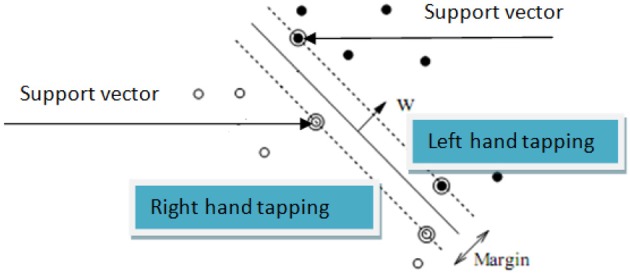
**Linear seperation hyperplane for right hand tapping feature and left hand tapping**.

In order to estimate hand tapping tasks, after determining coefficients of hand tapping times using the PR algorithm, we also used the linear SVM algorithm (Shawe-Taylor, 2000) to validate the coefficient data. In the linear SVM algorithm, assume that the training data are {*x*_*i*_, *y*_*i*_}, *i* = 1, …, l, *y*_*i*_ ∈ {−1, 1},*x*_*i*_ ∈ *R*^*d*^. The points *x* which lie on the hyperplane satisfy w.x + *b* = 0, in which | *b* |/∥ *w* ∥ is the distance from the hyperplane to the origin (where ∥ *w* ∥ is the Euclidean norm of *w*). Let *d*_+_ (*d*_−_) be the shortest distance from the seperation hyperplane to the closest positive (negative) samples corresponding to the coefficients of LH tapping and RH tapping, respectively. This is showed in Figure 7.

Margin of the hyperplane is *d*_+_ + *d*_−_. In the linear case, the support vector looks for the separating hyperplane with the largest margin using the primal Lagrangian. Suppose that all training data satisfy the following contraints:

(17) $$x_{i}\cdot w+b\geq +1,\text{ for }y_{i}=+1$$

(18) $$x_{i}\cdot w+b\leq -1,\text{ for }y_{i}=-1$$

The optimization problem is considered to transform Equations 17 and 18 using the primal Lagrangian as follows:

(19) $$L_{p}(w,b,\alpha )=\frac{1}{2}||w|{|}^{2}-\sum_{i=1}^{l}\alpha_{i}y_{i}(x_{i}\;\cdot \;w+b)+\sum_{i=1}^{l}\alpha_{i}$$

where α_*i*_ ≥ 0 are the Lagrange multipliers.

Differentiating *L*_*p*_ with respect to *w* and *b* and then getting the results to zeros, we have the following equation:

(20a) $$\frac{\partial L_{p}(w,b,\alpha )}{\partial w}=w-\sum_{i=1}^{l}y_{i}\alpha_{i}x_{i}=0$$

(20b) $$\frac{\partial L_{p}(w,b,\alpha )}{\partial b}=\sum_{i=1}^{l}y_{i}\alpha_{i}=0$$

Equations can be re-written to calculate the support vector as follows:

(21) $$w=\sum_{i=1}^{l}y_{i}\alpha_{i}x_{i}$$

The regressed data will trained using the SVM method, in which the hyperplane is a linear function and divided into two planes: D_+_ contains the coefficients and *y* = +1 is of the left tapping; similarly D_−_ has the coefficients and *y* = −1 is of the right tapping.

## Results and discussion

**Figure 8:**
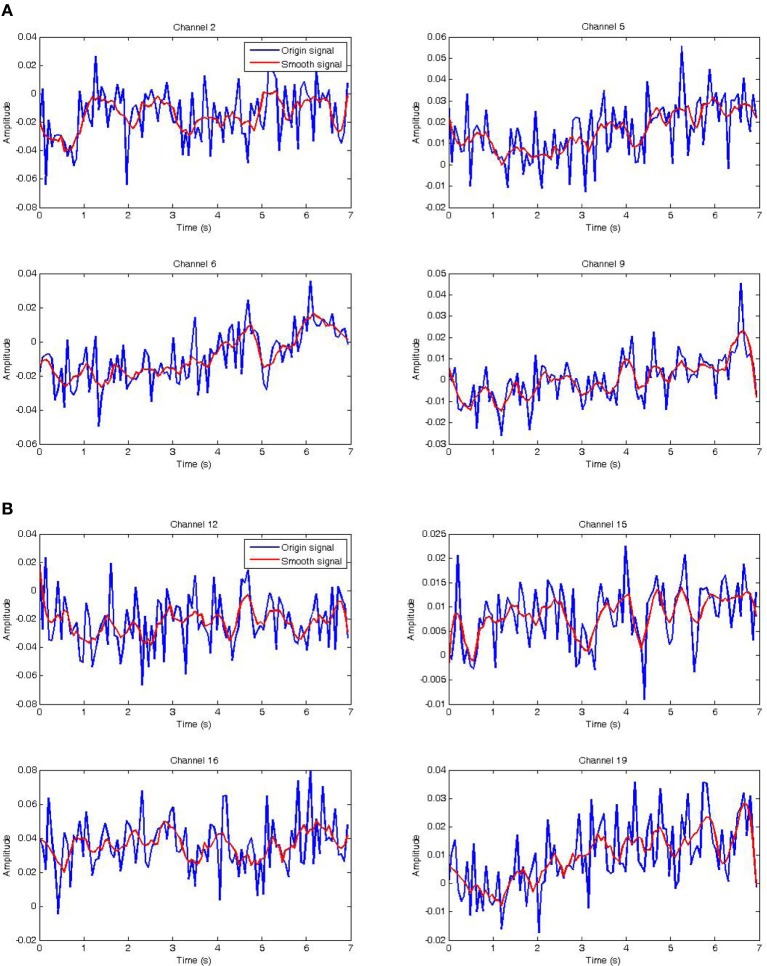
**Raw data and its smoothed version. (A)** Raw and smoothed NIRS data of channel 2, 5, 6, 9 of the left hemisphere. **(B)** Raw and smoothed NIRS data of channel 12, 15, 16, 19 of the right hemisphere.

**Figure 9:**
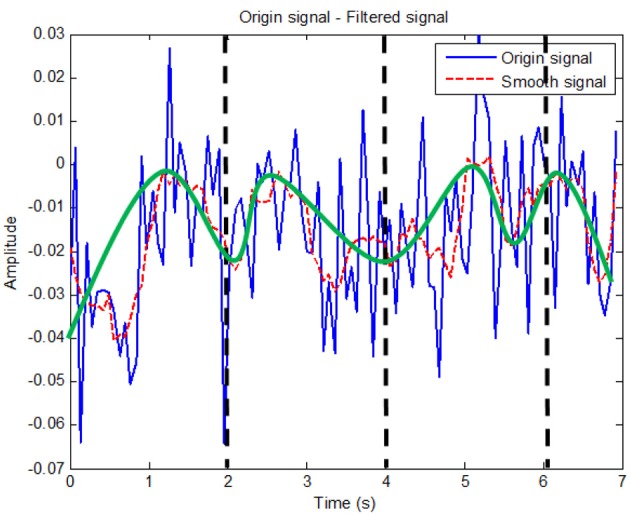
**Smoothed signal analysis**. Black dash line shows tapping time period while the green solid curve shows the changes in theory for channel 2.

Oxy-Hb raw signals (blue) were collected from the fNIRS system using the proposed protocol (see Figure 5) which plays an important role during measure tasks. In particular, each subject tapped his hand up or down 10 times in 20 s. Therefore, we could separate this task into 10 parts, in which each part has 1 s up and 1 s down as shown in Figure 9. Before analyzing Oxy-Hb signals, the Savitzky–Golay (SG) filter was chosen to produce the smooth Oxy-Hb signals (red) as shown in the Figures 8A,B. In this filter, if the size of the window is too small, noises still affect upon the Oxy-Hb signals. Otherwise, if the large window size is chosen, the useful information may be lost. As mentioned before, Oxy-Hb signals using fNIRS technique are the concentration of Oxy-Hb in blood flow of human brain related to excitations or activities of human body. Therefore, choosing the window size as well as the order of the filter is very important and also depends on each typical case. For this reason, the SG with the window size of 11 and the order of 3 was applied (see Figures 8A,B).

After smoothing Oxy-Hb signal by using the SG filter, the features of Oxy-Hb signal corresponding to hand tapping are extracted using a PR algorithm. In this case, the PR algorithm with the order-5 polynomial produces six coefficients and its equation is represented as follows:

(22) $$y=h_{5}x^{5}+h_{4}x^{4}+h_{3}x^{3}+h_{2}x^{2}+h_{1}x+h_{0}$$

where *x* represents time from 0 to 7 s with the resolution of 0.07.

The fact is that choosing the window size as well as the order of the polynomial plays an important role due to avoidance of loosing information of signals. In Figures 8, 9, the red Oxy-Hb signals are the smoothed signals, in which the window size and the order were carefully calculated and chosen so that the peaks of the signals removed after filtering do not affect consequences on the analysis.

**Figure 10:**
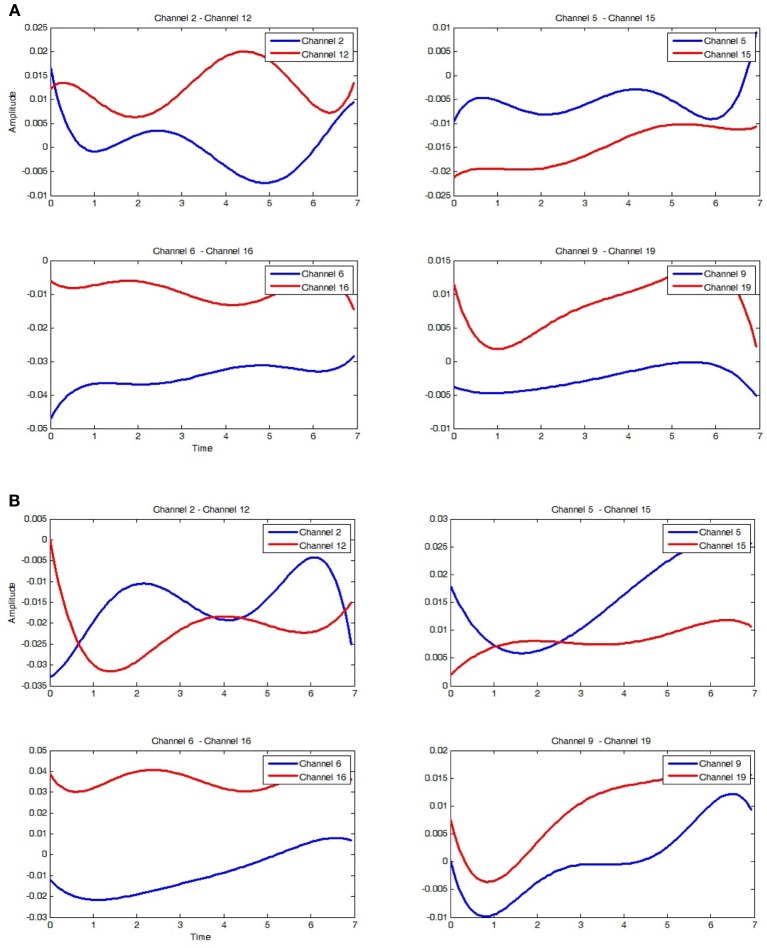
**Regressed polynomial corresponding to hand tapping. (A)** Regressed polynomial of 8 channels of the left hand tapping. Blue curves are the signals on the left brain side and red curves are of the right brain side. **(B)** Regressed polynomial of 8 channels of right hand tapping. Blue curves are the signals on the left brain side and red curves are of the right brain side.

This equation was applied to determine the regressed Oxy-Hb signals of the channels 2, 5, 6, 9 (left hemisphere) and the channels of the right hemisphere, 12, 15, 16, 19. Thus, the obtained results of the RH tapping and the LH tapping as showed in Figures 10A,B are compared together. However, these features of the Oxy-Hb signals obtained at two hemispheres are very hard to distinguish between are the right tapping and the left tapping. For this reason, training data, which are coefficients of the regressed polynomials as shown in Table 1, were applied to identify hand tapping tasks. In particular, in each time of hand tapping, Oxy-Hb concentration changes of two hemispheres allow us obtain the regressed coefficients using the PR algorithm. Moreover, six coefficients of each channel as shown in Table 2 are arranged to be a vector. For classification of hand tapping tasks, the vector was employed to the algorithms such as the ANN or SVM for training data.

**Table 2:** **The arrangement of the regressed coefficients of hand tapping tasks for obtaining the input of the recognition networks**.

**Left hand tapping coefficients**				**Right hand tapping coefficients**			
**Ch-2**	**Ch-5**	**Ch-6**	**Ch-9**	**Ch-12**	**Ch-15**	**Ch-16**	**Ch-19**
*h*_21_ … *h*_26_	*h*_51_ … *h*_56_	*h*_61_ … *h*_66_	*h*_91_ … *h*_96_	*h*_121_ … _126_	*h*_151_ … _156_	*h*_161_ … _166_	*h*_191_ … *h*_196_

Assume that *v*_*r*_ is the vector of the RH tapping and the vector of the LH tapping is *v*_*l*_. In one run of experiment, the subject performed a hand tapping task 20 times, in which 10 times for the LH tapping and 10 times for the RH one. Therefore, a set of the LH tapping coefficients *S*_*l*_ includes 10 vectors (from *v*_*l*1_ to *v*_*l*10_) and that of the RH tapping coefficients *S*_*r*_ is 10 vectors (from *v*_*r*1_ to *v*_*r*10_). With 80 sample vectors obtained from subjects, the recognition algorithm was worked out by splitting the sample vectors to be 4 runs of 20-fold cross recognition. For identifying the LH tapping, one used 9 vectors of the *S*_*l*_ set combined with the 10 vectors of the *S*_*r*_ set and the remaining vector of the *S*_*l*_ set is used to be a sample vector for identification. In the case of identifying the RH tapping, 9 vectors of the *S*_*r*_ set combined with the 10 vectors of the *S*_*l*_ and the remaining one is used to be the sample vector for identifying. As known, Oxy-Hb signals obtained from human brain have many noises and artifacts. Therefore, identifying hand tapping tasks corresponding to Oxy-Hb concentration changes is not easy. For this reason, the identification algorithms such the ANN and SVM are reliable in this research. In the SVM method, the linear hyperplane was chosen. In each training process, the values α (having 15 values of α) are produced, also there are 15 support vectors *w* (each vector *w* is 48 elements) and *b* is 0.068. In similarity, the ANN algorithm with the hidden layer of 100 nodes was applied to obtain the training result, in which the goal of training is set up of 0.001 and the number of epochs is 5000.

From the data sets of the hand tapping tasks, the SVM algorithm was applied for learning to analyze data and recognize patterns. In understanding this SVM training algorithm, data vectors from the hand tapping tasks are given the input of the classifier with a hyperplane which forms two possible classes of the output. In this method, experimental results to the LH tapping of Subject-1 and Subject-3 are the same and Subject-2 showed the lower performance with just 72.5% of the accuracy compared with 82.5% of Subject-1 and Subject-3 as shown in Table 3. While basically its results are the same to that of tapping the RH side.

**Table 3:** **Experiment result of 3 subjects with SVM**.

**Run**	**Hand tapping**	**Accuracy—subject 1 (%)**	**Accuracy—subject 2 (%)**	**Accuracy—subject 3 (%)**
1	Right	70	80	80
	Left	80	80	70
2	Right	90	80	80
	Left	100	70	90
3	Right	90	60	90
	Left	80	70	100
4	Right	70	80	80
	Left	70	70	70
Average	Right	80	75.0	82.5
	Left	82.5	72.5	82.5

The ANN algorithm used for identifying hand tapping tasks here consists of one input layer, one hidden layer of 100 nodes and the output layer with two nodes. In addition, the second method in this paper is one of recognition methods which have been applied in recent years. Although this algorithm is used very popular for recognition problems, it still uses here due to giving the good performances and also being a reliable method. The result is that classification using the ANN method gave the around 83% performance of tapping the RH side is higher than the performance of around 73% for the RH tapping as shown in Table 4.

**Table 4:** **Experiment result of 3 subjects with ANN**.

**Run**	**Hand tapping**	**Accuracy—subject 1 (%)**	**Accuracy—subject 2 (%)**	**Accuracy—subject 3 (%)**
1	Right	80	80	70
	Left	70	80	60
2	Right	90	90	80
	Left	80	70	80
3	Right	90	70	100
	Left	70	70	80
4	Right	80	90	80
	Left	70	80	70
Average	Right	85	82.5	82.5
	Left	72.5	75	72.5

All the results of hand tapping tasks obtained here have the accuracy of more than 70%. In this research, two methods were applied to find the best one. The first method is that the SVM algorithm is used for recognition on three subjects and produce different performances. In particular, Subject-3 with tapping the RH has the best result with over 80% of the accuracy, while the accuracy of Subject-2 is only 75% for the case of the RH tapping and 72.5% for that of the LH tapping. While the second method using the ANN is that the accuracy in the case of the RH tapping is equal to or greater than 82.5%. In particular, Subject-1 has the best accuracy of the right tapping, while the accuracy of the left tapping just stops at 72.5% for both Subject-1 and Subject-3. Moreover, the result is that Subject-2 has the best accuracy in the case of the LH tapping. It is clear that two methods used in this research give a little bit different performance. In general, the SVM method is better in this case. We also observed that Subject-2 produced the best accuracy in the case of the LH tapping. The right tapping accuracy is greater than the left tapping of all three subjects in the case of the SVM is performed. Each classification network has a different response to the same inputs. It can give the good accuracy in some cases of the right tapping, but it can show the poor in others. Because of this selective problem, one should more carefully choose the classification network type to obtain the higher performance.

In recent years, researchers have proposed different algorithms in exploring body activities related to human brain. The poor spatial resolution of NIRS made it difficult to distinguish two closely located cortical areas from each other. A combination of the multi-channel NIRS and a Center of Gravity (CoG) approach widely accepted in the field of Transcranial Magnetic Stimulation (TMS) could be used to discriminate between closely located cortical areas activated during hand and foot movements of the subject (Koenraadt et al., 2012). Hemodynamic responses were measured using a NIRS system of 8 channels. For estimating adapt of Oxyhemoglobin (OHb) and Deoxyhemoglobin (HHb), a CoG algorithm was determined for each condition using the mean hemodynamic responses and the coordinates of the channels. Therefore, significant hemodynamic responses were found for hand and foot movements. This is the interesting methods which can be applied to develop for identifying hand tapping. Based on this information, the proposed algorithms in our research can be improved with some thresholds to find out which channel gives the valuable information. The order of the filter we had chosen here belongs to the pulses time of moving hand up and down. Thus, the method to quantitatively estimate the start and end timing of the hand movement using the neural network was proposed.

In (Muroga et al., 2006), the authors measured regional cerebral blood flow during tapping movement of the RH using NIRS technique. The following tendencies of total-Hb were observed, in which Hb increased within 10 s from the movement start time, decreased within 10 s from the movement end time. The direction of arm force from hemoglobin concentration changes measured by using NIRS technique was discriminated. A Self-Organizing Map (SOM) was used to classify the force direction information obtained from the NIRS signals. The results confirmed that the direction of the arm force is discriminable through the NIRS signal. In the simple classification approach, the average discrimination rate gave the performance of 87.5% for two directions. The experimental results showed that the NIRS signal from arm force contained information related to the force directions (Sato et al., 2009). This research is from our research about the proposed methods and experimental tasks. While the SOM method, possibly called the ANN, evaluated the arm force directions with the 87.5% large performance is a little bit higher than that compared with the SVM and the ANN for the LH and RH tapping tasks. This is one of methods which we need to apply for our experimental tasks to determine the best one.

In using NIRS technology, the local distribution of fingers (right thumb and ring finger, respectively) was distinguished to hemodynamic responses on Somatosensory cortex by the electrical stimuli intensity (SI), whose results showed in good accordance with the anatomical arrangement of hand area (Xu et al., 2007). Another application is that in NIRS-based brain activation mapping, a novel real-time NIRS signal analysis framework based on the General Linear Model (GLM) and the Kalman estimator was proposed (Ge et al., 2010). A set of simulated data was processed using the proposed framework. The results obtained suggested that the method can effectively locate brain activation areas in real-time, thereby demonstrating its potential for real-time NIRS-based brain imaging applications. Both these researches, the authors were proposed the same experiment with finger movements using different methods. It is clear that the NIRS technology is not only used to distinguish hand tapping tasks in this paper, but also applied for finger movement tasks.

From the previous researches, we have realized that the proposed algorithm can be accompanied with other algorithms for finding more accuracy. The NIRS technology has been used to obtain Oxy-Hb signals in recent years. However, these Oxy-Hb signals always exist noises and artifacts due to subject movements, noisy environments, human biological changes and others. Proposing a good method for estimating Oxy-Hb concentration changes related to brain activities is always necessary to researchers. In particular, the poor resolution in spatial domain needs to be overcome and also applications in real time are an interesting field for research developments using the NIRS technology.

## Conclusion

In this paper, original brain signals of hand tapping tasks were filtered by the Savitzky–Golay filter to produce the smooth signals. Moreover, the smoothed signals of the LH and RH tapping tasks corresponding to Oxy-Hb concentration changes in human brain were analyzed using the PR algorithm. Based on different coefficients of the curves obtained from the PR algorithm, the ANN and SVM algorithms were employed to validate Oxy-Hb data for the recognition of the hand tapping times. Experimental results with hand tapping times showed that one could distinguish the LH or RH tapping tasks of the subject. In addition, from the obtained results of two methods, it was realized that the SVM algorithm is faster than the ANN one in term of time recognition. Based on the proposed algorithms, future work is that experiments will be developed on many subjects to investigate more accuracy and to apply for treatment and rehabilitation.

### Conflict of interest statement

The authors declare that the research was conducted in the absence of any commercial or financial relationships that could be construed as a potential conflict of interest.
